# Caloric restriction as a possible pitfall for persistent acromegaly follow-up – case report

**DOI:** 10.1186/s12902-023-01319-0

**Published:** 2023-03-27

**Authors:** Ana Filipa Martins, Mónica Santos, Francisco Rosário

**Affiliations:** 1 Endocrine Department, Hospital da Luz de Lisboa, Av Lusíada, Nr 100, 1500-650 Lisbon, Portugal; 2 Nutrition Department, Hospital da Luz de Lisboa, Av Lusíada, Nr 100, 1500-650 Lisbon, Portugal

**Keywords:** Persistent acromegaly, Caloric restriction, Oral Glucose Tolerance Test, PTGO

## Abstract

**Background:**

Acromegaly diagnosis is established when plasma levels of IGF-1 are increased and the Oral Glucose Tolerance Test (OGTT) with 75gr of glucose can’t suppress Growth Hormone (GH) levels. These two parameters are also useful during follow-up, after surgical/radiologic therapy and/or during medical therapy.

**Case presentation:**

A 29-year-old woman was diagnosed with acromegaly after a severe headache. Previous amenorrhea and facial and acral changes were noticed. A pituitary macroadenoma was found, biochemical evaluation was in agreement with the suspected acromegaly and a transsphenoidal adenectomy was performed. As the disease recurred, a surgical reintervention and radiosurgery (Gamma Knife, 22 Gy) were necessary. No normalization of IGF-1 was achieved during three years after radiosurgery. Surprisingly, then, and although clinical features seemed getting worse, IGF-1 levels became consistently controlled to 0.3–0.8 times the upper limit of the reference range. Questioned, the patient referred that she was following an intermittent fasting dietary plan. However, based on the dietary questionnaire, she was found to be under severe caloric restriction. First OGTT (under caloric restriction) showed absence of GH suppression and an IGF-1 value of 234 ng/dL (Reference Range 76–286 ng/mL). A second OGTT, one month after an eucaloric diet was instituted, showed an increased IGF-1 of 294 ng/dL, maintaining an unsuppressed, yet less elevated, GH.

**Conclusions:**

GHRH/GH/IGF-1 axis controls somatic growth. Regulation is complex, and nutrition status and feeding pattern have a recognized role. Like systemic inflammation or chronic liver disease, fasting and malnutrition decrease the expression of hepatic GH receptors, with consequent reduction of IGF-1 levels, through resistance to GH. This clinical report shows that caloric restriction may represent a pitfall in acromegaly follow-up.

## Background

Acromegaly is a rare systemic disease caused by pathologic secretion of growth hormone (GH) and Insulin-like growth factor (IGF1). Recent population studies, mainly from Europe, estimate a prevalence of acromegaly between 2.8 and 13.7 cases per 100 000 people, similar between males and females in the majority of the studies. The annual incidence was estimated of 0.2 to 1.1 cases per 100 000/year [1, 2]. Patients were diagnosed in the median age that varied between 40.5 and 47 years [1].

Around 95% of all cases are due to pituitary adenoma [3] and at the time of diagnosis 2/3 are macroadenomas [1].

Less than 1% of cases are identified as familial, mainly due to MEN1 and FIPA [1].

A myriad of clinical features are recognized; among the most prevalent (> 60% of patients) are acral growth, deformity of orofacial features, soft tissue swelling, increased sweating, arthropathy, snoring syndrome and asthenia. Regarding mass effect symptoms, headache is the most frequent, identified in as many as 60% of patients [4, 5].

In 2014 the Endocrine Society published evidence-based guidelines focusing diagnosis, treatment and follow up of acromegaly [3]. These guidelines focus the determination of serum IGF-1 to rule out acromegaly in patients with suspicious clinical features, coexistence of several associated conditions/complications of acromegaly and/or in the diagnosis of a pituitary mass [3]. A cornerstone role is also posed in IGF-1 serum levels to monitor response to therapy and to define persistent vs controlled or cured acromegaly. However several conditions may interfere with IGF-1 determination, conditioning false positive and false negative results.

## Case presentation

A 29-year-old female was evaluated in the Emergency Department (ED) of our hospital in January 2011 because of an acute and severe headache since the previous day, which started in the end of a daytime shift as nurse.

Her past medical history included a congenital patent interauricular communication and rheumatic fever when she was 6. She had an uneventful pregnancy with labor two years earlier.

The CT scan performed identified a pituitary macroadenoma. Pituitary MRI better described a sellar lesion of 20 × 17x13mm, obliterating the suprasellar cistern and compressing the optic chiasm. A T2 hypersignal surrounding the right aspect of the lesion rose the possibility of a small hemorrhagic event or a cystic portion. Retrospectively, patient had been in amenorrhea for 5 months. Her rings were tighter and she increased two shoe numbers. She was also aware of dysmorphia of her nose which was wider.

Biochemical evaluation revealed normal results regarding glycaemia, prolactin, adrenal and gonadal axis; phosphocalcic metabolism was also within the reference range; IGF-1 was 4.4 times above the upper limit of the reference range (ULRR).

Patient was proposed for surgery and transsphenoidal adenectomy was performed with no immediate complications.

Three and a half months later her IGF-1 levels were normal (0.57 times the ULRR), in June 2011. Four months later she got pregnant. During pregnancy IGF-1 determinations were in the reference range, however, two months after labor, acromegaly recurred (IGF-1 1.5 times the ULRR), while breastfeeding. Sellar MNR suggested the presence of small residual tissue of the previous macroadenoma. Radiosurgery was proposed, however, patient expressed the desire of get pregnant again, what occurred 3 years later.

After labor in May 2016, RMN identified an ovalar macroadenoma of 15 × 11x17mm, with no invasion of neighbor structures. Again, patient was submitted to transsphenoidal surgery in January 2017 and, because of incomplete resection of this lesion, to radiosurgery (Gamma Knife, 22 Gy) in November 2018. After this procedure patient maintained eucortisolism and eugonadism, and oral contraceptive was started (desogestrel 0.075 mg daily).

**Fig. 1:**
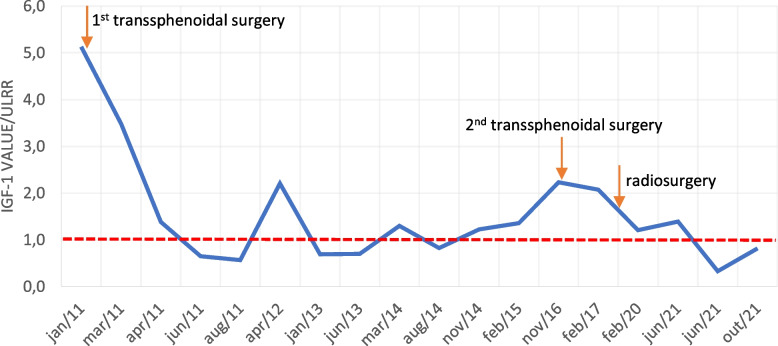
Evolution of IGF-1 since the diagnosis of acromegaly

IGF-1 remained elevated until 2021. However, after June 2021, IGF-1 systematically and consistently decreased to 0,3–0,8 times ULRR (Fig. 1), although worsening of clinical features was evident. When discussed with the patient, she explained she googled a new solution for acromegaly: intermittent fasting. Indeed, she was irregularly following an hypocaloric low carb diet (1263 kcal/day; 68% of her total energy estimated needs), composed by near 33% of carbs, 23% proteins and 43% lipids, distributed in 3 meals from 2:00 pm to 9:00 pm. This diet was intensified for periods.

Although reluctant, she accepted to perform an OGTT. As a result, a paradoxical increase in GH occurred, with a maximum value of 7.36 ng/mL at the 120 min of the test (Table 1). A new dietary inquiry found that during the week before OGTT she followed a very low caloric diet that included up to 20% of her energetic needs (378/1860 kcal), consisting in 32% of carbs (0.5 g/kg), 18% of proteins (0.3 g/kg) and 50% of lipids (0.4 g/kg), distributed in 3 meals from 2:00 pm to 9:00 pm.

**Table 1:** First 75-g OGTT results while patient was following a very low caloric diet (378/1860 kcal daily); RR, reference range

	0 min	30 min	60 min	90 min	120 min
Glucose (mg/dL)		132	106	60	73
GH (ng/mL)	1.38	3.46	5.05	6.73	7.36
IGF-1 (RR: 76–286 ng/mL)	234				

When OGTT was repeated, two weeks after an eucaloric diet of 1930 kcal/day (33 kcal/kg of body weight), consisting in 44% of carbs (3.6 g/kg), 14% of proteins (1.1 g/kg) and 42% of lipids (1.5 g/kg), a different response was observed (Table 2): basal IGF-1 was now increased and, although GH values remained suppressed after the patient drank the sugary solution, they were less elevated than before.

**Table 2:** Second 75-g OGTT results following two weeks of an eucaloric caloric diet (approximately 1930 kcal/day); RR, reference ange

	0 min	30 min	60 min	90 min	120 min
Glucose (mg/dL)	85	84	68	71	67
GH (ng/mL)	1.37	1.89	2.69	2.51	2.63
IGF-1 (RR: 76–286 ng/mL)	294				

## Discussion and conclusions

Although biggest country registries mainly from Europe show that medical therapies have gained space over the last years in the treatment of acromegaly (mainly for macroadenomas and invasive adenomas), even as first line therapy [2, 6–9], surgery still represents the best chance for cure. Concordantly, international guidelines and recommendations emphasize transsphenoidal surgery as the first line treatment of acromegaly [3, 10–12].

Gamma knife radiosurgery (GKS) is a treatment option for recurrent or persistent disease in patients with acromegaly. Patients achieving remission vary between 17 and 96% [13]. Remission rates after GKS increase in a time-dependent manner after GKS: Ronchi et al. documented 6%, 25% and 45% remission rates (with a GH nadir < 1 ng/ml and normal IGF-1 levels) after 3, 7 and 10 years after GKS, respectively [14] and Kong et al. reported 15%, 20.3% and 44.9% at 3, 5 and 10 years after GSK, respectively, but defining remission rates as GH levels < 2.5 ng/mL with normal age-adjusted IGF-1 [15]. Another group studied 110 patients who underwent GKS. After a mean follow-up time of 6.5 ± 4.7 years 16.4% were in remission and 23.6% were uncontrolled [16]. The mean time after GKS to remission was 26.5 months [16]. Taking this data into account, the patient we report could effectively be in remission 31 months after GKS. However, the clinical features didn’t match and were getting worse.

IGF-1 and GH determination are fundamental biochemical parameters used not only in the diagnosis, but also in the follow up of acromegaly. Criteria for remission of acromegaly suffered changes over the last two decades: a consensus statement published in 2000 suggested IGF-I levels within the normal range and nadir GH levels below 1 μg/L following OGTT to define cure [10]. 2010 consensus maintained the criteria of IGF-1 level within the reference range to define cure but reduced GH nadir following OGTT to 0.4 μg/L [11]. In 2014, Endocrine society recommended measurement of IGF-1 and random GH 3 months after surgery. GH nadir after OGTT < 0,14 μg/L was also recommended for patients whose random GH is greater than 1ug/L [3]. After radiation therapy, the last consensus also recommended annual GH/IGF-1 assessment following medication withdrawal to determine disease status [3].

Levels of IGF-1 depend on GH concentration in a log-linear relationship [17], so that normal levels of IGF-1 are assumed as effective to exclude diagnosis of acromegaly [3].

IGFs or somatomedins are the main effectors of GH [18] and both IGF1 and GH work together to promote longitudinal growth, as well as modulate metabolic function in adults [19].

Although IGF-1 serum levels are stable throughout the day [18], several conditions are recognized to increase and decrease results.

Conditions reducing IGF-1 levels in general population include hypothyroidism, poorly controlled diabetes mellitus, systemic inflammation, chronic liver and kidney diseases, oral estrogens, obesity, and prolonged fasting and malnutrition. While obesity is associated with low levels of GH, all the other conditions are usually characterized by GH increase, in a pattern of GH resistance [3, 18, 20, 21].

Fasting/Feeding and GH axis regulation are mutually dependent: nutritional status plays a key role in the regulation of GH secretion, and GH influences nutrients utilization and metabolism in humans and animals [22].

More recently, Caputo M. et al. [22], reviewed dietary nutrients and patterns impact on regulation of GH and IGF-I. Regarding acromegaly, they conclude that a) eucaloric very-low-carbohydrate ketogenic diet, b) periodical or prolonged regimens of caloric restriction, c) Okinawa diet (poor in proteins and rich in carbohydrates) and d) modified diets poor in leucine, valine and isoleucine, may favor acromegaly control. In the clinical setting, Coopmans et al. [23] published in 2020 a pivotal study focusing the impact of an eucaloric very-low-carbohydrate ketogenic diet (35 g of carbohydrate per day) as adjuvant to medical treatment in acromegaly. 11 patients with active disease under first-generation somatostatin receptor ligands were followed for 2-weeks. The authors aimed to reduce IGF-I synthesis following the down-regulation of hepatic growth hormone receptors through induction of ketosis and reduction of portal insulin concentrations. During the diet IGF-I concentration significantly decreased from 1.10 to 0.83 times the upper limit of the normal range, normalizing in all but one patient. Growth hormone did not increase during the two weeks. In half of the patients who maintained this diet after the study, dose reduction of somatostatin receptor ligand was possible [23].

Similarly, in the clinical case we describe, reduced IGF1 values and increased GH were observed during extreme caloric restriction. Then, when an eucaloric diet was introduced, IGF1 values increased above the reference range, while GH values remained elevated, albeit to a lesser extent. Relevant is the fact that, although the IGF1 values decreased during the relatively long period of caloric restriction (intermittent fasting and periods of severe restriction), no improvement was observed in relation to the clinical and phenotypic characteristics of acromegaly.

The case we report highlights fundamental aspects from clinical practice: first of all, the imperative need to contemplate all the conditions that may interfere with the correct judgment of analytic and functional tests our patients are submitted. Second, the possible traps for clinicians in the era of easy information that patients get on the internet, and they assume to know, ignoring the pitfalls behind. After the first OGTT, two scenarios were possible: our patient maintained disease, and medical therapy might be equated; or our patient was effectively in remission two and a half years after radiotherapy, but the severe caloric restriction of the previous week was responsible for the GH increase.

Only the second OGTT, under correct caloric intake, was informative: disease persistence was confirmed not only by the value of serum IGF-1, but also by the unsuppressed GH following 75gr of oral glucose. Indeed, blood glucose washout was faster in this second OGTT (considering the inferior peak glucose value—84 vs 132 mg/dL), compatible to higher basal insulin production.

As we focused, nutrition status interferes with the GH/IGF1 axis in different ways and may represent a therapeutic opportunity, but in this particular case it represented a pitfall in diagnosis and follow-up.

## Data Availability

All the data generated and/or analysed during this study are included in this published article.

## Abbreviations

CT: Computed tomography
ED: Emergency department
FIPA: Familial isolated pituitary tumors
GH: Growth hormone
GKS: Gamma knife radiosurgery
IGF-1: Insulin-like growing factor
MEN1: Multiple endocrine neoplasia
MNR: Magnetic nuclear resonance
OGTT: Oral glucose tolerance test
RR: Reference range
